# Supplementary material for: Dynamic network biomarker indicates pulmonary metastasis at the tipping point of hepatocellular carcinoma
Source: Nat Commun. 2018 Feb 14;9:678. doi: 10.1038/s41467-018-03024-2 (PMC5813207; doi:10.1038/s41467-018-03024-2)
Supplement: Supplementary file 1 — Supplementary Information [file 41467_2018_3024_MOESM1_ESM.pdf]

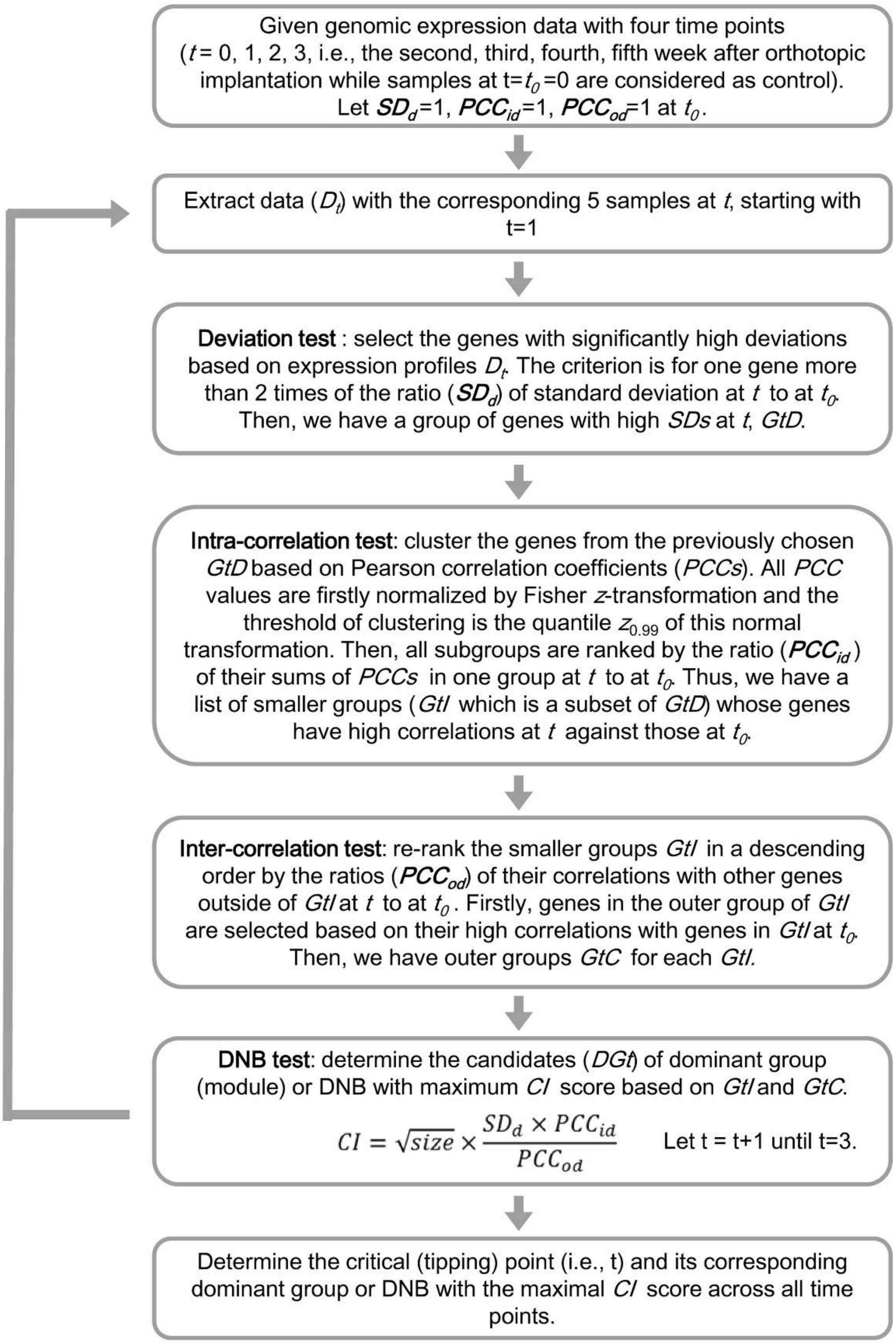

Supplementary Figure 1. The detailed algorithm of DNB method. A flowchart describes the detailed procedures to determine DNB members based on our mathematical method.

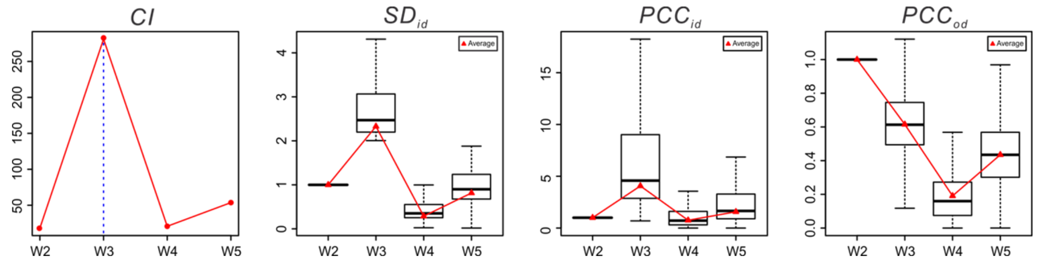

Supplementary Figure 2. Indices of DNB model during the progression of pulmonary metastasis of hepatocellular carcinoma. This series of diagrams visually show the comprehensive index *CI* and three key criteria of DNB after analyzing this whole genome-wide expression profile from the orthotopic xenograft mice at 4 well-designed time points. Note that, SD_{id}, PCC_{id} and PCC_{od} are calculated after compared with the corresponding control ones.

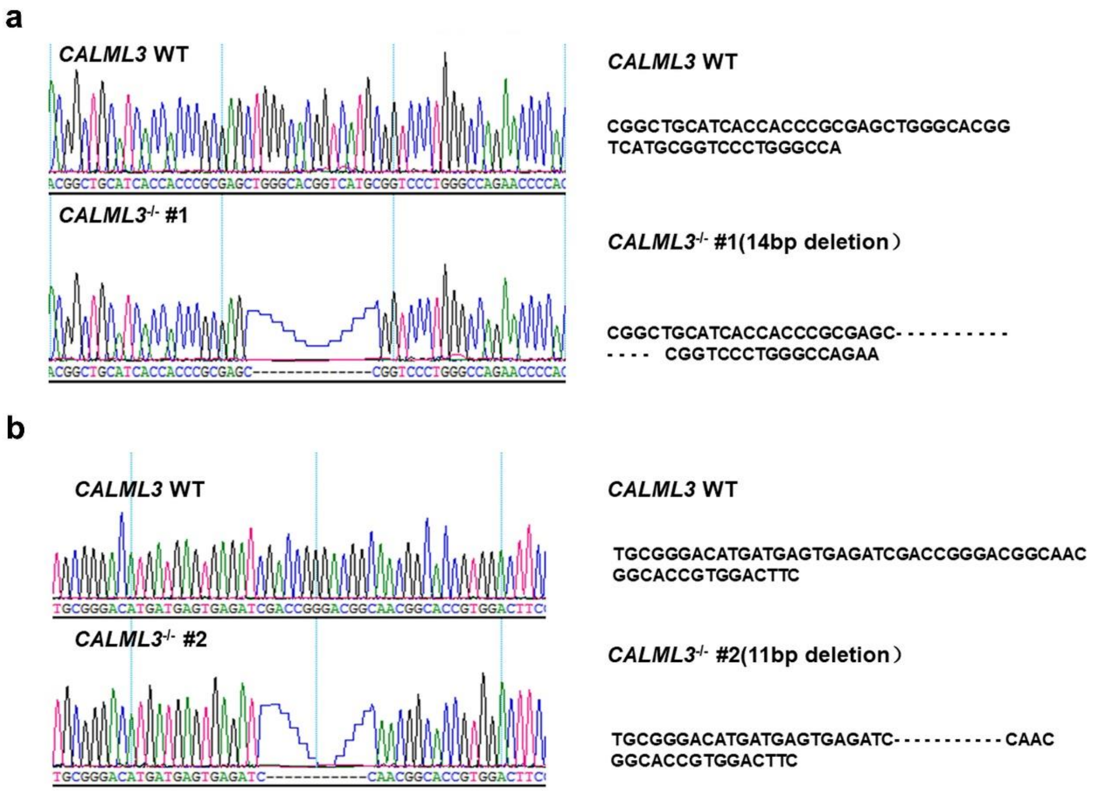

Supplementary Figure 3. Individual colonies (CALML3$^{-/-}$ #1 and CALML3$^{-/-}$ #2 cells) of CALML3 knockout by CRISPR/Cas9 were genotyped by genomic DNA sequencing. (**a**) CALML3$^{-/-}$ #1 cells had deletions of 14bp in CALML3 genomic region. (**b**) CALML3$^{-/-}$ #2 cells had deletions of 11bp in CALML3 genomic region. WT, wild type.

.

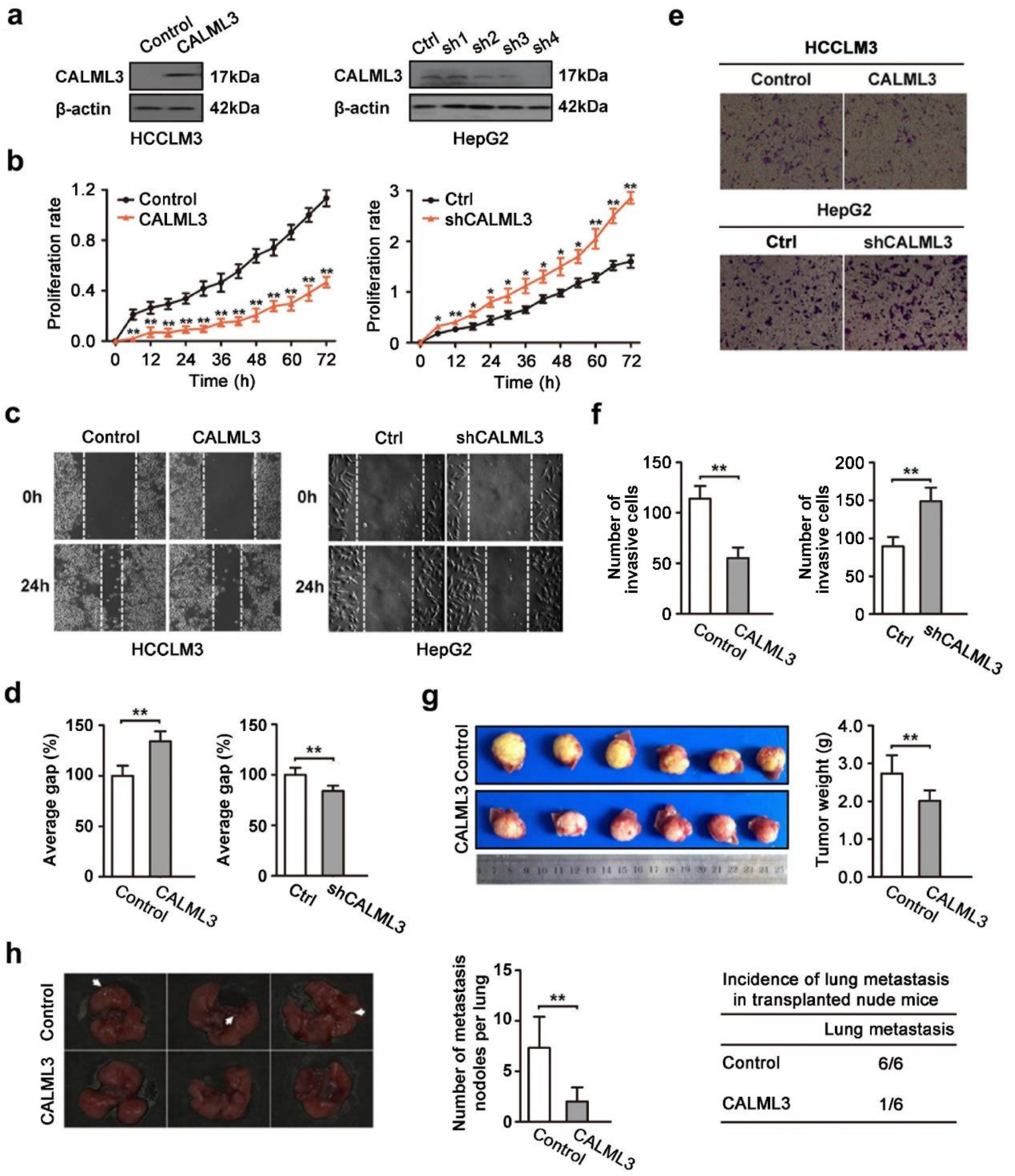

Supplementary Figure 4. CALML3 could suppress HCC metastasis *in vitro* and *in vivo.* (**a**) The efficiency of CALML3 overexpression in HCCLM3 cells and knockdown in HepG2 cells by lentivirus mediated expression and RNA interference was tested by Western blot. Of four different clones of CALML3 knockdown, sh4 produced the best knockdown efficiency and was chosen for further functional assays named shCALML3. (**b**) CALML3 overexpression suppressed cell proliferation in HCCLM3 cells while CALML3 knockdown promoted cell proliferation in HepG2 cells. (**c**) CALML3 overexpression suppressed cell migration (left panel) while CALML3 knockout brought the opposite effect (right panel). (**d**) Quantification of migration cells in the indicated groups in the wound healing assay.

CALML3 overexpression showed obvious suppression of migration abilities in HCCLM3 cells while CALML3 knockdown induced significant promotion of migration abilities in HepG2 cells. (**e**) CALML3 overexpression suppressed cell invasion while CALML3 knockdown brought the opposite effect. (**f**) Quantification of invasive cells in the indicated groups in the transwell invasion assay. (**g**) Tumours from mice implanted with HCCLM3 cells (the control and CALML3 overexpression cells). CALML3 overexpression showed less tumour weight. (**h**) Lung tissues from mice implanted with HCCLM3 cells (the control and CALML3 overexpression) orthotopic transplantation model were examined by fluorescence microscopy (Left panel). CALML3 overexpression HCCLM3 cells showed less lung metastatic nodules (Middle panel) and reduced incidence of lung metastasis (Right panel). Experiment was independently repeated three times. Student t-test. $*P < 0.05$, $**P < 0.01$. Error bars in panels are defined by s.d. (standard deviation).

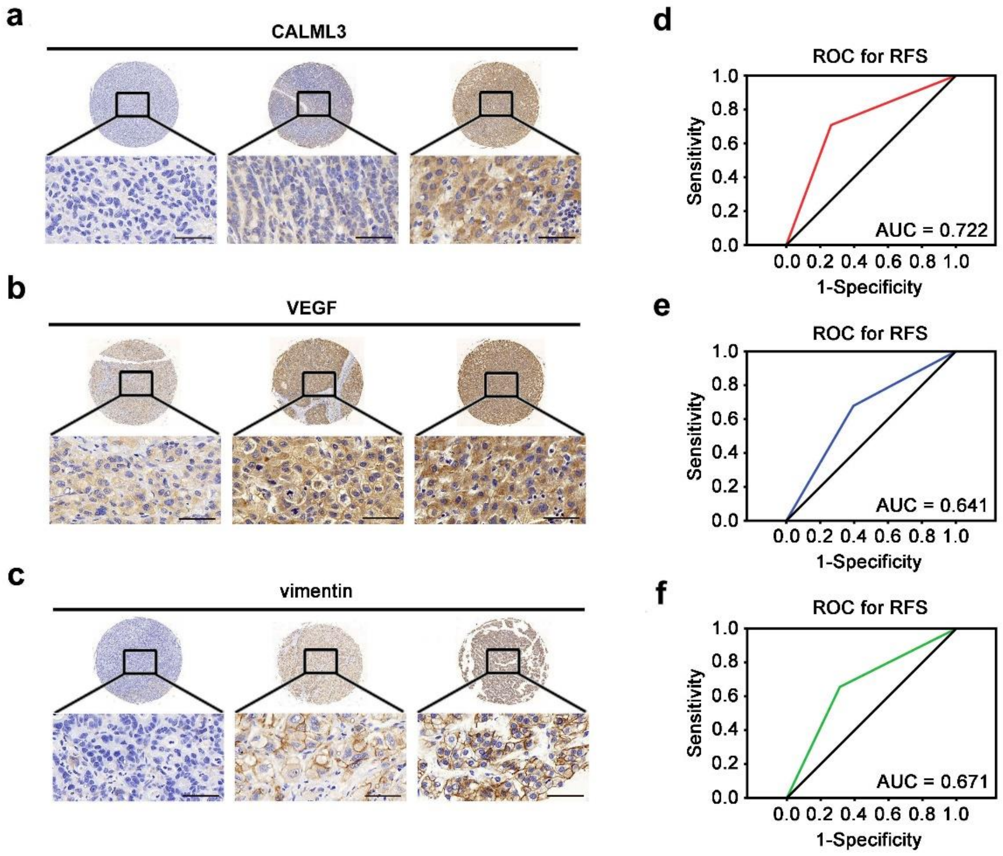

Supplementary Figure 5. The predictive ability of CALML3 compared with VEGF and vimentin by receiver operating characteristic (ROC) curves for relapse-free survival (RFS). (**a-c**) Typical immunochemistry staining of CALML3 (**a**), VEGF (**b**) and vimentin (**c**) in tumour tissues of HCC patients. (**d-f**) ROC analysis of CALML3 (**d**), VEGF (**e**) and vimentin (**f**) for RFS.

Original blots for Fig. 3b

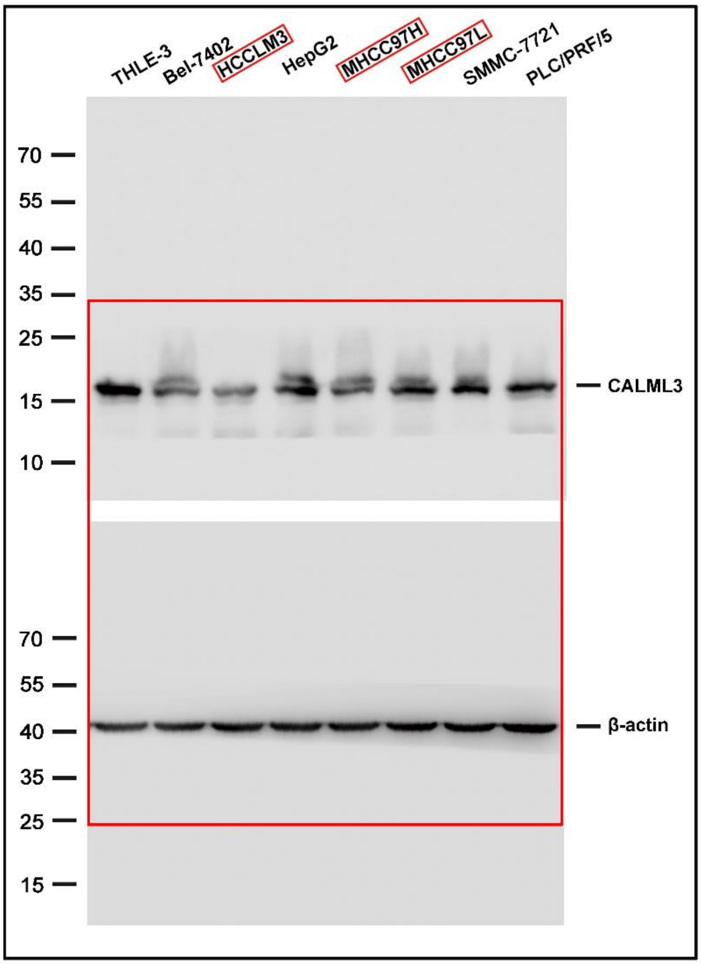

Supplementary Figure 6. Uncropped images of immunoblots.

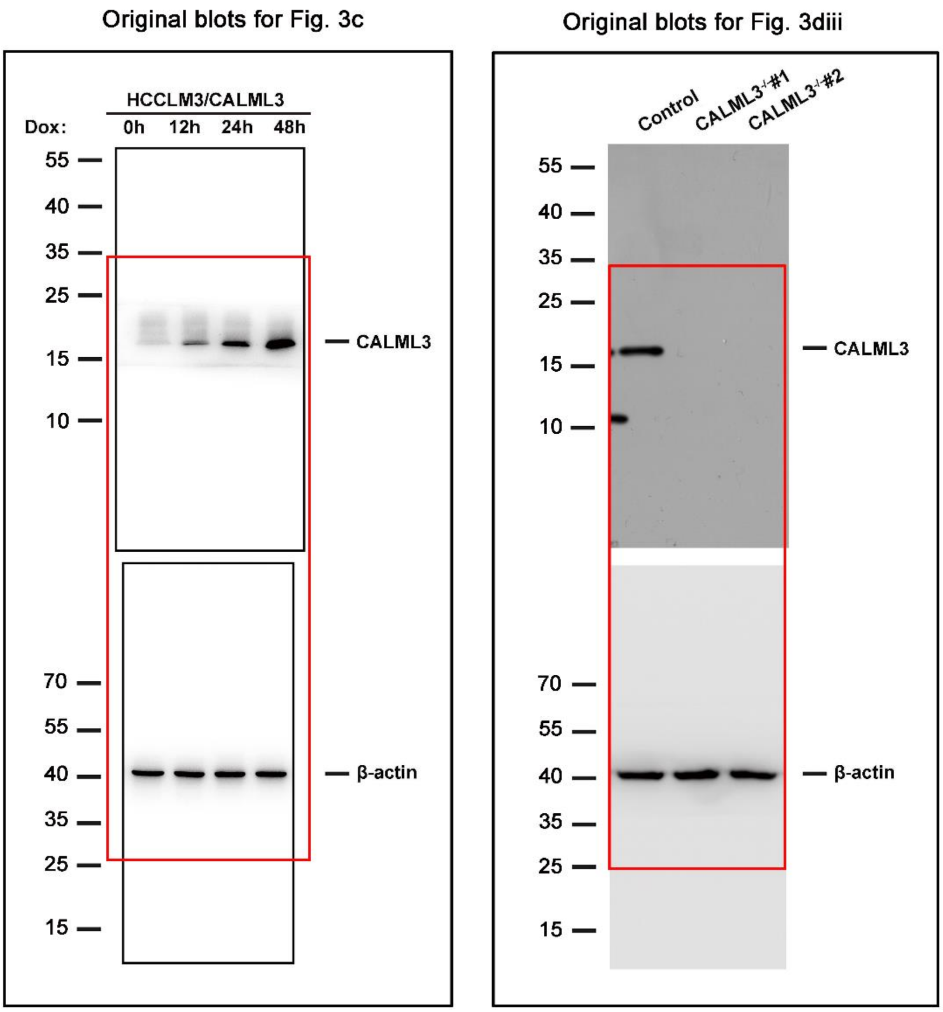

Supplementary Figure 7. Uncropped images of immunoblots.

Supplementary Table 1. The clinicopathological characteristics of 270 HCC patients enrolled in the study.

Clinical and pathological indexes		Non-pulmonary Metastasis Group (n=170)		pulmonary Metastasis Group (n=100)		*P* value
		n	%	n	%	
Gender	Female	26	15.3	13	13	0.605
	Male	144	84.7	87	87	
Age, years	≤ 52	86	50.1	56	56	0.390
	> 52	84	49.9	44	44	
Hepatitis B surface antigen	Negative	145	85.3	85	85	0.948
	Positive	25	14.7	15	15	
Liver cirrhosis	No	116	68.2	71	71	0.643
	Yes	54	31.8	29	29	
Tumor encapsulation	None	69	40.6	58	58	**0.006**
	Complete	101	59.4	42	42	
Vascular invasion	No	111	65.3	49	49	**0.009**
	Yes	59	34.7	51	51	
Tumor number	Single	144	84.8	78	78	0.164
	Multiple	26	15.2	22	22	
Tumor size, cm	≤ 5	81	47.6	40	40	0.222
	> 5	89	52.4	60	60	
Edmondson grade	I-II	126	74.1	65	65	0.112
	III-IV	44	25.9	35	35	
BCLC stage	A	47	27.6	16	16	**0.029**
	B/C	123	72.4	84	84	
ALT,U/L	≤ 75	154	90.6	84	84	0.106
	>75	16	9.4	16	16	
TB,μmol/L	≤ 20	149	87.6	90	90	0.558
	> 20	21	12.4	10	10	
Albumin,g/l	≤ 35	10	5.9	10	10	0.212
	>35	160	94.1	90	90	
PT, s	≤ 13	141	82.9	65	65	**0.001**
	>13	29	17.1	35	35	
AFP, ng/mL	≤ 20	69	40.6	37	37	0.560
	> 20	101	59.4	63	63	
CALML3 in tumor tissue	Negative	70	41.2	86	86	**<0.001**
	Positive	100	58.8	14	14	

ALT, alanine aminotransferase; BCLC, Barcelona Clinic Liver Cancer; TB, total bilirubin; PT, prothrombin time; AFP, alpha fetoprotein; OS, overall survival; RFS, relapse-free survival. Bold values indicate $P < 0.05$, P values from χ^2 test.

Supplementary Table 2. Univariate analyses of factors associated with overall survival (OS) and relapse-free survival (RFS)

Clinical and pathological indexes	OS Hazard ratio(95％CI)	OS *P* value	RFS Hazard ratio(95％CI)	RFS *P* value
Gender (Female vs. Male)	1.300 (0.757-2.233)	0.341	0.994 (0.655-1.508)	0.976
Age, years (≤ 52 vs. > 52)	1.091 (0.765-1.555)	0.631	0.988 (0.733-1.331)	0.937
Hepatitis B surface antigen (Negative vs. Positive)	0.735 (0.444-1.218)	0.232	0.839 (0.552-1.273)	0.409
Liver cirrhosis (No vs. Yes)	1.056 (0.711-1.570)	0.786	1.019 (0.735-1.412)	0.911
Tumor encapsulation (None vs. Complete)	0.550 (0.384-0.789)	**0.001**	0.632 (0.468-0.852）	**0.003**
Vascular invasion (No vs. Yes)	1.931 (1.354-2.752)	**<0.001**	1.852 (1.372-2.500)	**<0.001**
Tumor number (Single vs. Multiple)	1.548 (1.029-2.329)	**0.036**	1.283 (0.889-1.852)	0.183
Tumor size, cm (≤ 5 vs. > 5)	1.709 (1.182-2.470)	**0.004**	1.472 (1.088-1.991)	**0.012**
Edmondson grade (I-II vs. III-IV)	1.496 (1.032-2.169)	**0.034**	1.602 (1.169-2.196)	**0.003**
BCLC stage (A vs. B/C)	2.669 (1.609-4.427)	**<0.001**	1.472 (1.023-2.117)	**0.037**
ALT,U/L (≤ 75 vs. >75)	1.651 (1.022-2.667)	**0.041**	1.204 (0.769-1.883)	0.417
TB,μmol/L (≤ 20 vs. > 20)	1.716 (1.062-2.776)	**0.028**	1.106 (0.700-1.746)	0.667
Albumin,g/l (≤ 35 vs. >35)	0.838 (0.438-1.601)	0.592	0.676 (0.397-1.150)	0.149
PT, s (≤ 13 vs. >13)	1.101 (0.717-1.690)	0.661	1.447 (1.031-2.030)	**0.033**
AFP, ng/mL (≤ 20 vs. > 20)	1.496 (1.022-2.188)	**0.038**	1.240 (0.911-1.686)	0.171
CALML3 in tumor tissue (Negative vs. Positive)	0.425 (0.284-0.636)	**<0.001**	0.384 (0.273-0.538)	**<0.001**

Cox proportional hazards regression model was used in univariate analysis. AFP, alpha fetoprotein; ALT, alanine aminotransferase; BCLC, Barcelona Clinic Liver Cancer; OS, overall survival; PT, prothrombin time; RFS, relapse-free survival; TB, total bilirubin. Bold values indicate $P < 0.05$, *P* values from Cox regression analysis.

Supplementary Table 3. Multivariate analyses of factors associated with overall survival (OS) and relapse-free survival (RFS)

Clinical and pathological indexes	Hazard ratio (95% CI)	*P* value
OS		
Tumor encapsulation (None vs. Complete)	0.622 (0.421-0.917)	**0.016**
Vascular invasion（No/Yes）	1.516 (1.030-2.229)	**0.035**
Tumor number (Single vs. Multiple)	1.391 (0.860-2.249)	0.179
Tumor size, cm (≤ 5 vs. > 5)	1.347 (0.842-2.156)	0.214
Edmondson grade（I-II/III-IV）	1.276 (0.871-1.871)	0.211
BCLC stage (A vs. B/C)	1.927 (1.033-3.596)	**0.039**
ALT,U/L (≤ 75 vs. >75)	1.337 (0.815-2.191)	0.250
TB,μmol/L (≤ 20 vs. > 20)	2.199 (1.321-3.658)	**0.002**
AFP, ng/mL (≤ 20 vs. > 20)	1.285 (0.851-1.938)	0.233
CALML3 in tumor tissue (Negative vs. Positive)	0.630 (0.413-0.960)	**0.032**
RFS		
Tumor encapsulation (None vs. Complete)	0.790 (0.580-1.075)	0.134
vascular invasion (No vs. Yes)	1.449 (1.056-1.988)	**0.022**
Tumor size, cm (≤ 5 vs. > 5)	1.362 (0.941-1.972)	0.101
Edmondson grade (I-II vs. III-IV)	1.348 (0.976-1.861)	0.070
BCLC stage (A vs. B/C)	0.916 (0.583-1.440)	0.703
PT, s (≤ 13 vs. >13)	1.420 (1.008-2.001)	**0.045**
CALML3 in tumor tissue (Negative vs. Positive)	0.465 (0.324-0.667)	**<0.001**

Note: Variables were adopted for their prognostic significance by univariate analysis ($P < 0.05$). AFP, alpha fetoprotein; ALT, alanine aminotransferase; BCLC, Barcelona Clinic Liver Cancer; OS, overall survival; PT, prothrombin time; RFS, relapse-free survival; TB, total bilirubin. Bold values indicate $P < 0.05$, *P* values from Cox regression analysis.

Supplementary Table 4. Correlation between CALML3 and clinicopathologic characteristics

Clinical and pathological indexes	CALML3 expression in tumor tissue		
	Negative	Positive	*P* value
Patients	156	114	
Gender			
Female	21	18	0.591
Male	135	96	
Age (years)			
≤52	81	61	0.797
>52	75	53	
Hepatitis B surface antigen			
Negative	23	17	0.969
Positive	133	97	
Liver cirrhosis			
No	42	41	0.112
Yes	114	73	
Tumor encapsulation			
No	86	41	**0.002**
Complete	70	73	
Vascular invasion			
No	81	79	**0.004**
Yes	75	35	
Tumor number			
Single	123	99	0.090
Multiple	33	15	
Tumor size (cm)			
≤5	61	60	**0.027**
>5	95	54	
Edmondson grade			
I-II	106	85	0.238
III-IV	50	29	
BCLC stage			
A	25	38	**0.001**
B/C	131	76	
ALT (units/L)			
≤75	136	102	0.565
>75	20	12	
TB,μmol/L			
≤ 20	137	100	0.980
> 20	19	14	
Albumin,g/l			
≤ 35	12	8	0.834
>35	144	106	
PT, s			
≤ 13	113	93	0.081
>13	43	21	
AFP, ng/mL			
≤20	54	50	0.123
>20	102	64	

AFP, alpha fetoprotein; ALT, alanine aminotransferase; BCLC, Barcelona Clinic Liver Cancer; OS, overall survival; PT, prothrombin time; RFS, relapse-free survival; TB, total bilirubin. Bold values indicate $P < 0.05$, P values from χ^2 test.

Supplementary Table 5. The ROC analysis of variables for recurrence

Variables	Recurrence	
	AUC	95%CI
CALML3	0.722	0.657 to 0.787
VEGF	0.641	0.572 to 0.711
vimentin	0.671	0.604 to 0.739

Abbreviations: ROC, receiver operating characteristic; AUC, area under the curve; 95% CI, 95% confidence interval; VEGF, vascular endothelial growth factor.
