# Supplementary material for: Dynamic network biomarker indicates pulmonary metastasis at the tipping point of hepatocellular carcinoma
Source: Nat Commun. 2018 Feb 14;9:678. doi: 10.1038/s41467-018-03024-2 (PMC5813207; doi:10.1038/s41467-018-03024-2)
Supplement: Supplementary file 3 — Description of Additional Supplementary Files [file 41467_2018_3024_MOESM3_ESM.pdf]

Description of Additional Supplementary Files

File Name: Supplementary Data 1
Description: Information on differentially expressed genes.

File Name: Supplementary Data 2
Description: Information on members of dynamic network biomarker.

File Name: Supplementary Data 3
Description: HCC-associated KEGG pathways.

File Name: Supplementary Data 4
Description: KEGG pathways involving CALML3 and its 53 inversing DEGs before and after the critical period of metastasis initiation.

File Name: Supplementary Data 5
Description: RT-PCR results.
